# Pioglitazone enhances collateral blood flow in ischemic hindlimb of diabetic mice through an Akt-dependent VEGF-mediated mechanism, regardless of PPARγ stimulation

**DOI:** 10.1186/1475-2840-8-49

**Published:** 2009-09-08

**Authors:** Federico Biscetti, Giuseppe Straface, Vincenzo Arena, Egidio Stigliano, Giovanni Pecorini, Paola Rizzo, Giulia De Angelis, Luigi Iuliano, Giovanni Ghirlanda, Andrea Flex

**Affiliations:** 1 Laboratory of Vascular Biology and Genetics, Department of Medicine, A Gemelli University Hospital, Catholic University School of Medicine, Rome, Italy; 2 Unit of Vascular Medicine & Mass Spectrometry Lab, Department of Internal Medicine, Sapienza University of Rome, Italy; 3 Department of Pathology, Catholic University School of Medicine, Rome, Italy; 4 Department of Infectious Diseases, A Gemelli University Hospital, Catholic University School of Medicine, Rome, Italy

## Abstract

**Background:**

Type 2 diabetes mellitus (T2DM) is commonly associated with both microvascular and macrovascular complications and a strong correlation exists between glycaemic control and the incidence and progression of vascular complications. Pioglitazone, a Peroxisome proliferator-activated receptor-γ (PPARγ) ligand indicated for therapy of type T2DM, induces vascular effects that seem to occur independently of glucose lowering.

**Methods:**

By using a hindlimb ischemia murine model, in this study we have found that pioglitazone restores the blood flow recovery and capillary density in ischemic muscle of diabetic mice and that this process is associated with increased expression of Vascular Endothelial Growth Factor (VEGF). Importantly, these beneficial effects are abrogated when endogenous Akt is inhibited; furthermore, the direct activation of PPARγ, with its selective agonist GW1929, does not restore blood flow recovery and capillary density. Finally, an important collateral vessel growth is obtained with combined treatment with pioglitazone and selective PPARγ inhibitor GW9662.

**Conclusion:**

These data demonstrate that Akt-VEGF pathway is essential for ischemia-induced angiogenic effect of pioglitazone and that pioglitazone exerts this effect via a PPARγ independent manner.

## Background

Diabetes mellitus is commonly associated with both microvascular and macrovascular complications such as coronary artery disease, cerebrovascular events, severe peripheral vascular disease, nephropathy and retinopathy [1]. Vascular function in diabetes has been studied extensively in both animal models and humans [2-4], and impaired endothelium-dependent vasodilatation has been documented as a consistent finding in animal models of diabetes induced by alloxan or streptozotocin [5,6]. Consistently, *in vivo *studies have confirmed that hyperglycemia directly induces endothelial dysfunction both in diabetic and healthy subjects [7]. Moreover an experimental animal model has shown a decreased ability of diabetic mice in restoring the blood flow and the capillarity density after hind-limb ischemia [8]. Thiazolidinedione derivatives (TZDs), such as pioglitazone, troglitazone and rosiglitazone, are indicated for therapy of type 2 diabetes mellitus (T2DM). They have been demonstrated to be effective alone or in combination with a sulfonylurea, metformin, or insulin. Pioglitazone is an insulin sensitizer that promotes glucose metabolism without increasing insulin secretion [9]. In addition to its insulin sensitizing effects, increasing evidence suggests that this drug improve vascular health, vascular function and inflammatory biomarkers of arteriosclerosis [10-12]. Interestingly, these vascular effects seem to occur independently of glucose lowering and have been demonstrated also in non-diabetic, healthy individuals [10,12-14]. These findings have led to the hypothesis that pioglitazone could exert vasculoprotective effects that are independent of its metabolic action.

Peroxisome proliferator-activated receptors (PPARs) are the major ligands of TZDs and Peroxisome proliferator-activated receptor-γ (PPARγ) is the receptor mediating TZDs' antidiabetic effects [15]. TZDs are non-selective and non-specific ligands of PPARs and they are able to stimulate several PPARγ-independent pathways [16-21]. Therefore, the vasculoprotective effect of pioglitazone could be unrelated to the activation of PPARγ.

Akt is a central signaling molecule in regulating cell survival, proliferation, tumor growth and angiogenesis [22]. Short-term Akt activation in inducible Akt1 transgenic mice induces physiological cardiac hypertrophy with maintained vascular density [23], indicating that coronary angiogenesis is enhanced to keep pace with the growth of the myocardium. Similar observations have also been made in skeletal muscle cells: Akt activation results in myofiber growth associated with enhanced Vascular Endothelial Growth Factor (VEGF), a prototypical angiogenic agent, secretion and induces blood vessel recruitment [24]. VEGF and Angiopoietin-2 (Ang-2) are key angiogenic growth factors induced by hypoxia [25], and expression of these two growth factors is enhanced by short-term Akt activation in the myocardium [23]. Furthermore, transgenic co-expression of VEGF and Ang-2 exhibits synergistic effects on induction of coronary angiogenesis in the myocardium [26]. Thus, Akt-mediated growth-promoting signals act to enhance angiogenesis in a paracrine manner, providing a mechanism by which angiogenesis is coordinately regulated. Some authors have shown that the treatment with pioglitazone in an experimental model of hind-limb ischemia in diabetic mice up-regulates VEGF expression and this is associated with the phosphorylation/activation of eNOS at Ser^1177 ^and Akt at Ser^473 ^[8]. Given pre-existing data, we hypothesized that pioglitazone could improve impaired angiogenesis in diabetic mice by Akt-VEGF pathway, independently of PPARγ receptor.

## Methods

### Animals and drugs administration

The investigation was approved by A. Gemelli University Hospital Institutional Animal Care and Use Committee. Male 8-12-week-old C57BL/6J mice were used for experiments. Diabetes was induced by administering 50 mg/kg body wt streptozotocin (STZ; Sigma) in citrate buffer (pH 4.5), intraperitoneally (i.p.) during the fasting state, for 5 days, as previously described [27]. Hyperglycemia was verified 2 days after STZ injection by an Accu-Check Active glucometer (Roche). We considered mice to be diabetic when blood glucose was at least 16 mmol/l (normal 5-8 mmol/l). Overall, 50 mice showed a blood glucose level of at least16 mmol/l, both 1 and 2 week after the STZ injection and, therefore, were included in the experimental diabetic group. A first group of 10 STZ-diabetic mice received pioglitazone (3 mg/kg per day) by gavage for two weeks [8]. To evaluate whether the effect of pioglitazone was mediated by PPARγ, a second group of 10 STZ-diabetic pioglitazone-treated mice, received a PPARγ selective antagonist, GW9662 (Sigma), at the dosage of 2 mg/kg i.p., administered 1 h before pioglitazone [28]. To evaluate whether the effect of pioglitazone was mediated by Akt, a third group of 10 STZ-diabetic pioglitazone-treated mice, FPA-124 (Echelon), a cell-permeable inhibitor of Akt (Sigma), was administered by gavage at the dosage of 20 mg/kg/bid, everyday of pioglitazone-treatment period [29,30]. Finally, to evaluate the role of PPARγ on ischemia-induced angiogenesis, a fourth group of 10 STZ-diabetic mice was gavaged twice daily for two weeks with GW1929 (Sigma), a PPARγ selective agonist, at 5 mg/kg dosage [31]. The last group of 10 STZ-diabetic mice received vehicle (0.5% carboxymethyl cellulose) by gavage everyday for two weeks after surgery. Finally, 10 untreated C57BL/6J mice were also included in the model. The last two groups were used as controls.

### Mouse hindlimb ischemia model

After two weeks from the beginning of the treatment with pioglitazone (n = 10), pioglitazone + GW9662 (n = 10), pioglitazone + FPA-124 (n = 10), GW1929 (n = 10), unilateral hindlimb ischemia was induced by excising the right femoral artery, as previously described [32]. Right femoral artery ligation was used to induce hindlimb ischemia in untreated and the STZ-diabetic mice receiving vehicle. Briefly, all animals were anesthetized with an i.p. injection of ketamine (60 mg/kg) and xylazine (8 mg/kg). The proximal and distal portions of the femoral artery and the distal portion of the saphenous artery were ligated. The arteries and all side branches were dissected free and excised. The skin was closed with 5-0 surgical suture. A laser Doppler perfusion imager system (PeriScan PIM II, Perimed) was used to measure hindlimb blood perfusion before and after surgery and then followed at 7-day intervals, until the end of the study, for a total follow-up of 28 days after surgery [32]. Before imaging, excess hairs were removed from the limbs using depilatory cream and mice were placed on a heating plate at 40°C. To avoid the influence of ambient light and temperature, results were expressed as the ratio between perfusion in the right (ischemic) versus left (non-ischemic) limb.

### Histological Assays

At one and four weeks after surgery, mice were sacrificed by i.p. injection of an overdose of pentobarbital. The whole limbs were fixed in methanol overnight. The femora were carefully removed, and the ischemic thigh muscles were embedded in paraffin. All the specimens were routinely fixed overnight in 4% buffered formalin and embedded in paraffin. The sections for immunohistochemistry were collected on 3-aminopropyltriethoxy-silane (Sigma), allowed to dry overnight at 37°C to ensure optimal adhesion, dewaxed, rehydrated, and treated with 0.3% H2O2 in methanol for 10 min to block endogenous peroxidase. For antigen retrieval (CD31 only, not necessary for VEGF) the section were microwave treated in 1 mM EDTA at pH 8 for 10 min and allowed to cool for 20 min. Endogenous biotin was saturated using a biotin blocking kit (Vector Laboratories). The sections were incubated at room temperature for 30 min with the following antibodies: purified rat anti-mouse CD31 [dilution 1:30; monoclonal (IgG2a); BD Bioscience] and rabbit anti-mouse VEGF [dilution 1:100, polyclonal, Santa Cruz Biotechnology]. The slides were incubated for 1 h in the humid chamber at room temperature, then with peroxidase-conjugated secondary antibodies for 10 minutes, washed, incubated with DAB and counterstained with hematoxylin. Capillary density was measured by counting six random high-power (magnification × 200) fields or a minimum of 200 fibers from each ischemic and non-ischemic limb on an inverted light microscope, and was expressed by the number of CD31^+ ^cells per square millimeter or per fiber. Area was measured with a NIH Image analysis system (ImageJ 1.41). Two operators extracted independently the results.

### Western Blotting

Immunoblotting was performed on homogenates of muscle tissues. Proteins (40 mg per lane) were separated in 10% SDS-polyacrylamide gels and transferred onto polyvinylidine difluoride membranes [33]. Membranes were incubated with antibodies against phospho-Akt (Ser473, 1:500, Cell Signaling Technology Company), Akt (1:1000, New England Biolabs) and VEGF (1:500, R&D Systems). Antibody binding was detected with horseradish peroxidase-conjugated secondary antibodies (1:2000; Chemicon) and enhanced chemiluminescence system (GE Healthcare Bioscience). Finally, the blots were reprobed with total Akt, VEGF, or actin (1:5000, Sigma).

### Statistics

All data are expressed as mean value ± SEM. Statistical comparisons of means were performed by ANOVA followed by Student's t-test. A *p *value of < 0.05 was considered to be statistically significant.

## Results

### Pioglitazone enhances blood flow recovery in diabetic mice after hind-limb ischemia

**Figure 1:**
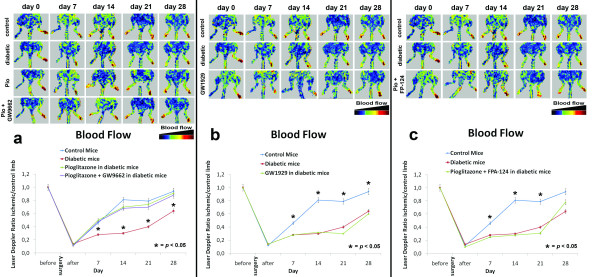
**a. Foot blood flow monitored *in vivo *by laser Doppler perfusion imaging (LDPI) in control, STZ-diabetic, pioglitazone-treated and pioglitazone+GW9662-treated mice**. Evaluation of the ischemic (right) and non-ischemic (left) hindlimbs, immediately after and on days 7, 14, 21 and 28 after surgery. Red indicates normal perfusion while blue a marked reduction in blood flow of ischemic hindlimb. Pioglitazone restored blood flow recovery in diabetic mice, compared with normoglycemic mice. Interestingly, pioglitazone associated with selective PPARγ inhibitor GW9662 restored blood flow recovery in diabetic mice. The blood flow of the ischemic hindlimb is expressed as the ratio between perfusion of the ischemic limb versus uninjured limb. *p < 0.05 vs control, STZ-diabetic and pioglitazone-treated mice. **b**. LDPI in control, STZ-diabetic, GW1929-treated mice. Selective PPARγ agonist GW1929 did not restore blood flow recovery in diabetic mice, compared with normoglycemic and STZ-diabetic mice.*p < 0.05 vs STZ-diabetic and GW1929-treated mice. **c**. LDPI in control, STZ-diabetic and pioglitazone+FP-124-treated mice. Pioglitazone associated with selective Akt inhibitor FP-124 did not restore blood flow recovery in diabetic mice, compared with normoglycemic and STZ-diabetic mice.*p < 0.05 vs STZ-diabetic and pioglitazone+FP-124-treated mice.

**Figure 2:**
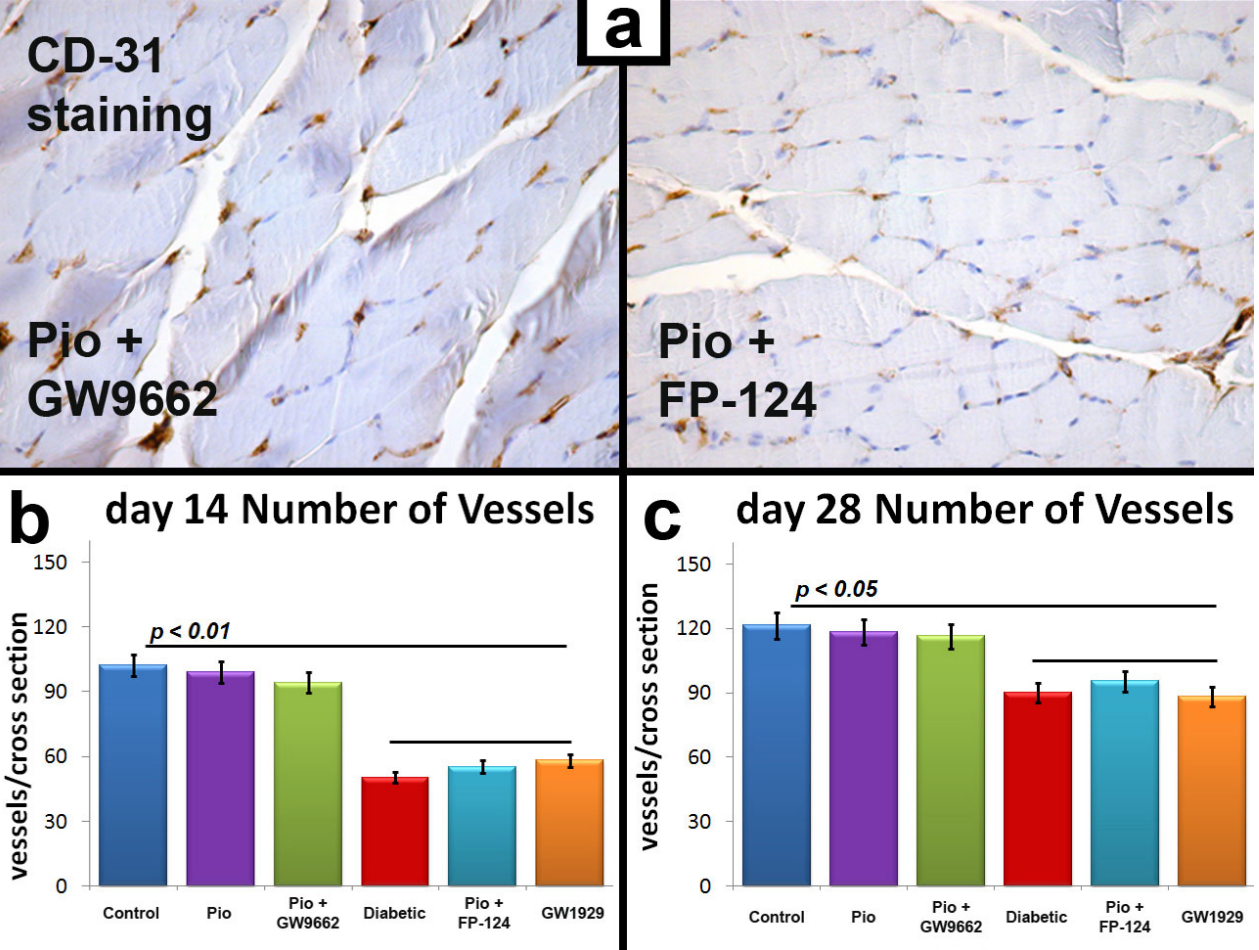
**a. Representative photomicrographs of ischemic muscle sections from pioglitazone+GW9662-treated and from pioglitazone+FP-124-treated diabetic mice stained with antibody directed against CD-31, 28 days after surgery**. Positive staining appears in brown. Magnification ×40. Number of vessels per cross section is significantly reduced in pioglitazone+FP-124-treated diabetic mice respect to GW9662-treated diabetic mice. **b**. Quantification of ischemia-induced angiogenesis 14 and 28 days after surgery. Number of vessels per cross section is significantly reduced in STZ-diabetic, pioglitazone+FP-124-treated and GW1929-treated mice vs STZ-diabetic, pioglitazone+FP-124-treated and GW9662-treated mice (*p *< 0.01 at 14 days and *p *< 0.05 at 28 days).

Pioglitazone treatment results in improved perfusion and favorably modulation of capillary density in ischemic skeletal muscle of diabetic mice. Laser Doppler perfusion imaging was performed before, immediately after and on days 7, 14, 21 and 28 after surgery. Perfusion recovery was significantly attenuated in STZ-diabetic mice treated with vehicle, compared with normoglycemic mice (Fig. 1a) and, as previously reported [8], pioglitazone restored the blood flow recovery in STZ-diabetic mice, reaching almost 80% of the blood flow of the untreated leg in four weeks (Fig. 1a). Collateral vessel formation was also histologically evaluated by the capillary density of the ischemic hindlimb muscle collected two and four weeks after surgery (Fig. 2). Consistently with the measurement of laser Doppler imaging, anti-CD31 immunostaining revealed that angiogenesis in the ischemic hindlimb is impaired in the diabetic mice treated with vehicle. Pioglitazone significantly restored the number of detectable capillaries in the ischemic leg of the diabetic mice to a normal level (Fig. 2).

### Pioglitazone-induced angiogenic response occurs in association with VEGF production and increases phosphorylation of Akt

**Figure 3:**
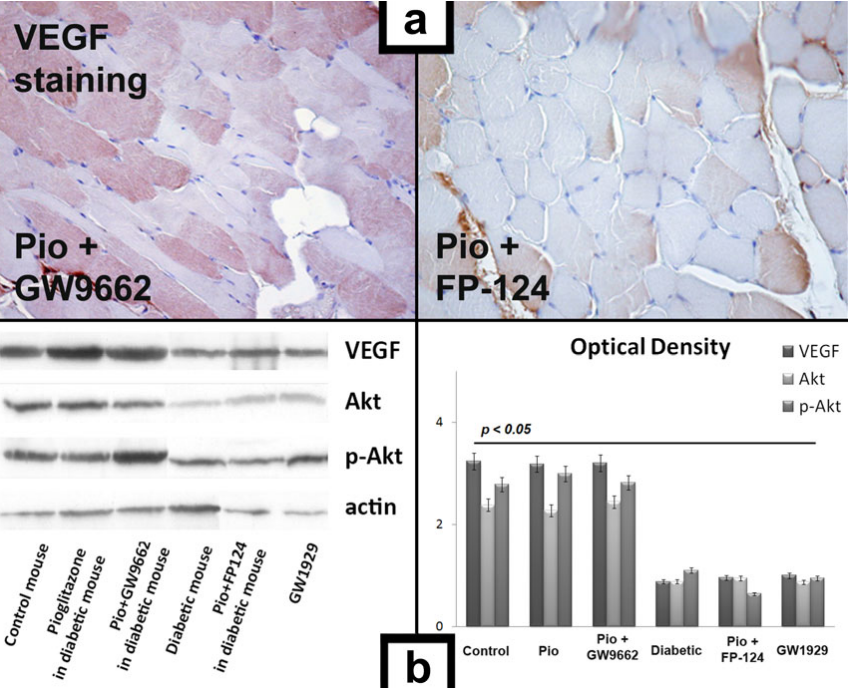
**a. Representative photomicrographs of ischemic muscle sections from pioglitazone+GW9662-treated and from pioglitazone+FP-124-treated diabetic mice stained with antibody directed against VEGF, 7 days after surgery.** Positive staining appears in brown. Magnification ×40. **b**. VEGF, Akt and pAkt expression 7 days after surgery. Representative Western blot and optical density evaluation of VEGF Akt, pAkt and actin protein content in the ischemic legs of control, pioglitazone-treated, pioglitazone+GW9662-treated, STZ-diabetic, pioglitazone+FP-124-treated and GW9662-treated mice. Pioglitazone normalized VEGF expression with enhanced phosphorylation of Akt in ischemic muscle. After pioglitazone administration, even when PPARγ activity was inhibited by GW9662, STZ-diabetic mice showed a normalized expression of VEGF and enhanced levels of p-Akt Ser473 in ischemic limbs compared to those treated with pioglitazone+FP-124 or with GW1929. Selective PPARγ agonist GW1929 or pioglitazone associated with selective Akt inhibitor FP-124 did not normalize VEGF expression in diabetic micevs STZ-diabetic, pioglitazone+FP-124-treated and GW1929-treated mice. *p *< 0.05.

Immunostaining revealed that VEGF expression had increased in the ischemic tissue of normoglycemic mice compared to STZ-diabetic mice (Fig. 3). VEGF concentration in ischemic tissue was also significantly higher in pioglitazone-treated STZ-mice than in untreated STZ-diabetic mice (Fig. 3), underlying the crucial role of VEGF in pioglitazone-induced angiogenic response. To further investigate the mechanism by which pioglitazone stimulates angiogenesis in diabetic mice, we evaluated VEGF and Akt expression in the ischemic leg 7 days after surgery by Western blotting analysis (Fig. 3). Pioglitazone normalized VEGF expression and induced phosphorylation/activation of Akt at Ser^473^, which expression was reduced in diabetic mice treated with vehicle.

### The ability of pioglitazone in normalizing the expression of VEGF, activating Akt and restoring blood flow is independent of PPARγ activation

To determine if pioglitazone-induced angiogenic response in STZ-diabetic mice depends on activation of PPARγ, we compared two groups of STZ-diabetic mice undergoing hindlimb ischemia with selective PPARγ agonist GW1929 (n = 10) or with pioglitazone associated with selective PPARγ inhibitor GW9662 (n = 10). Laser Doppler perfusion imaging was performed before, immediately after and on days 7, 14, 21 and 28 after surgery. Surprisingly, the treatment with GW1929 did not exert any effect on ischemic muscle (Fig. 1b), on VEGF expression (Fig. 3) and on blood flow recovery, while pioglitazone maintained its activity in normalizing the expression of VEGF (Fig. 3), activating Akt and restoring blood flow also in mice where PPARγ was inhibited by GW9662 (Fig. 1a).

These results suggest that pioglitazone presides its favorable effects on ischemic-induced angiogenesis regardless of PPARγ activity.

### The activity of pioglitazone on VEGF expression and blood flow recovery was abolished by selective inhibition of Akt

To confirm the hypothesis that the up-regulation of VEGF induced by pioglitazone occurs through the activation of Akt pathway, we inhibited Akt in diabetic mice subjected to hind-limb ischemia and pre-treated with pioglitazone. Seven days after surgery Akt was inhibited and pioglitazone had no effect on VEGF expression of ischemic muscles in diabetic mice (Fig. 3). Furthermore, combined treatment of pioglitazone with FPA-124, a selective Akt inhibitor, had no effect on blood flow recovery evaluated by laser Doppler (Fig. 1c).

## Discussion

In this study, we have investigated the effect of pioglitazone on angiogenesis in response to tissue ischemia in diabetic mice. We have been able to show that vascular Akt system plays an important role in ischemia-induced angiogenesis in pioglitazone-treated mice *in vivo*. In fact, pioglitazone failed to promote blood flow recovery when Akt activity was inhibited by FP-124, indicating that Akt is an essential co-factor to promote collateral growth in response to tissue ischemia during treatment with pioglitazone. To the best of our knowledge, this is the first study demonstrating the important roles of vascular Akt system in ameliorating endothelial dysfunction and restoring ischemia-induced angiogenesis in diabetic mice treated with pioglitazone, including induction of post-ischemic angiogenesis and secretion of VEGF from ischemic muscle. In addition, this study demonstrates that the positive effect of pioglitazone occurs via a PPARγ independent mechanism.

T2DM is a leading cause of morbidity and mortality that places a substantial economic and health burden on the public. Although the increased death rate is mainly due to cardiovascular disease, deaths from non-cardiovascular causes are also increasing [34]. T2DM is commonly associated with both microvascular and macrovascular complications [35]. A strong correlation exists between glycaemic control and the incidence and progression of microvascular complications [36], while its impact on macrovascular events evolution seems weaker [37]. Several long-term diabetes mellitus complications are characterized by vasculopathy associated with aberrant angiogenesis. Excessive angiogenesis plays an important role in diabetic retinopathy, nephropathy and neuropathy whereas inhibited angiogenesis contributes to impaired wound healing and impaired coronary collateral vessel development. Indeed, diabetic neuropathy is linked to reduced nutritive blood flow secondary to diabetes and could potentially be improved by inducing angiogenesis in regions of inadequate perfusion [38]. Furthermore, the increased glomerular filtration rate in diabetic nephropathy may be the consequence of an enlarged glomerular filtration surface resulting from excessive angiogenesis [39]. Moreover, diabetic patients frequently suffer of chronic non-healing ulcers usually localized on pressure points of the foot [40] and the presence of small abnormal blood vessels has been reported at the wound edge of diabetic ulcers [41]. The increased morbidity and mortality related to atherosclerosis and the ensuing coronary and peripheral artery disease may be due to an impaired ability to form collateral vessel in the diabetic scenario [42]. Indeed, these patients often present a wide vascular disease and a great number of vascular occlusions, due to diabetes-induced deficiencies of angiogenesis [43]. Diabetes-induced impairment of collateral formation has been also demonstrated in murine models: hindlimb ischemia created by ligation of the femoral artery is associated with a reduced formation of capillaries and a reduction in blood flow to the ischemic limb in diabetic versus non-diabetic mice [44]. Thus, several agents are implicated in development of abnormal angiogenesis in diabetes mellitus, including dysregulation of VEGF local expression [45,46] and other growth factors or cytokines imbalances [47,48] in a general metabolic derangement [49].

PPARs are considered important factors for ameliorating hyperlipidemia and hyperglycemia in subjects with T2DM. PPARγ activation improves insulin sensitivity, decreases inflammation, plasma levels of free fatty acids and blood pressure, so indirectly leading to inhibition of atherogenesis, improvement of endothelial function and reduction of cardiovascular events. The insulin-sensitizing drugs TZDs, as PPARγ agonists, have beneficial effects on serum lipids in diabetic patients and have also been shown to inhibit the progression of atherosclerosis in animal models [17]. Increasing evidence suggests that TZDs improve endothelium-dependent vascular function and inflammatory biomarkers of arteriosclerosis, independently of glucose-lowering effect in diabetic and non-diabetic individuals [50]. Interestingly, TZDs were reported to increase VEGF expression in human vascular smooth muscle cells [51], to promote angiogenesis after focal cerebral ischemia [52] and to reduce myocardial infarction size in animal model [53]. Recent findings showed that pioglitazone restores the impaired angiogenesis in ischemic muscle of diabetic mice [8]. In all these studies, Authors have assumed that the effects induced by TZDs were mediated by the activation of PPARγ, without considering that TZDs are non-selective and non-specific ligands of this nuclear receptor, since they are able to stimulate several PPARγ-independent pathways that are important in angiogenesis [18-21,54]. About pioglitazone, there are many biological effects idipendent of PPARγ activation; in fact, pioglitazone causes PPARγ-independent relaxation of isolated blood vessel [55], inhibits homocysteine-induced vascular smooth muscle cells migration that is independent of PPARγ [56] and prevents apoptosis of endothelial progenitor cells (EPCs) in mice as well as in human EPCs in a PI3K-dependent manner [57]. Furthermore, this agent inhibits lung cancer cell growth and its antiproliferative action does not occurs via PPARγ activation [58]. Importantly, pioglitazone is metabolized predominantly via the CYP 3A4, CYP 2C8 and CYP 1A1 pathways, through hydroxylation and oxidation [59] and, since the CYP 3A4 metabolic pathway is common to the metabolism of several drugs, the potential for drug interactions and consequent alterations in efficacy and safety of numerous concomitant medications should be considered when this drug is co-administered with CYP 3A4-metabolized agents. Effectively, pioglitazone stimulates PPARγ but the vasculoprotective effects could be unrelated to the activation of PPARγ.

In a hindlimb ischemia murine model, our results confirm that diabetic mice display a decreased angiogenic response, that pioglitazone restores the blood flow recovery and capillary density in ischemic muscle and that this process is associated with increased expression of VEGF. Importantly, these beneficial effects are abrogated when endogenous Akt is inhibited; furthermore, the direct activation of PPARγ, with its selective agonist GW1929, does not restore blood flow recovery and capillary density. Finally, an important collateral vessel growth is obtained with combined treatment with pioglitazone and selective PPARγ inhibitor GW9662. These data demonstrate that Akt pathway is essential for ischemia-induced angiogenic effect of pioglitazone and that pioglitazone exerts this effect via a PPARγ independent manner.

The epidemic of T2DM has created a large need for new hypoglycaemic and hypolipemic therapies; the PPARs agonists represent a potentially important new group of drugs with a mechanism of action differing from and perhaps complementary to existing therapies. The emergence of the angiogenesis altered signalling paradigm in T2DM promises to enhance our understanding of cardiovascular complications of diabetes and the advances in the understanding of the biology of angiogenesis enabled the development of new therapeutic strategies for promoting angiogenesis. Since pioglitazone is a drug currently used with excellent tolerance and limited toxicity, our data might offer a novel and potentially low toxic approach for the treatment of diabetes-associated altered angiogenesis. Our findings provide new information to understand the biological effects of pioglitazone and its role in ischemia-induced angiogenesis, with potentially important implications for the management of subjects affected by T2DM cardiovascular complications.

## Conclusion

The novel finding of the present study is that vascular Akt system plays an essential role in restoring angiogenesis in diabetic mice treated with pioglitazone. In fact, pioglitazone failed to promote blood flow recovery when Akt activity was abolished, indicating that Akt is essential for pioglitazone to promote collateral growth in response to tissue ischemia. Finally, this is the first demonstration that the positive effect of pioglitazone occurs via a PPARγ independent mechanism. This study represents a step forward towards a better understanding of mechanisms implicated in the vascular effects of pioglitazone.

## Competing interests

The authors declare that they have no competing interests.

## Authors' contributions

FB and GS participated in the design of the study, performed the hindlimb ischemia model and in part performed data analysis. VA and ES performed the immunohistochemical analysis. PR and GP carried out the immunoassays. GDA performed the statistical analysis. LI reviewed the manuscript. GG and AF conceived of the study, and participated in its design and coordination and helped to draft the manuscript. All authors read and approved the final manuscript.
